# Metformin-Induced Lactic Acidosis Following Intentional Overdose: A Case Report and Mini Review

**DOI:** 10.7759/cureus.97415

**Published:** 2025-11-21

**Authors:** Muhammad Usman Taj, Mohammed S Mohammed, Muhammad Asif Jan, Hind A Sirag, Adnane Guella

**Affiliations:** 1 Intensive Care Unit, University Hospital Sharjah, Sharjah, ARE; 2 Internal Medicine, University Hospital Sharjah, Sharjah, ARE; 3 Nephrology, University Hospital Sharjah, Sharjah, ARE

**Keywords:** dialysis, dialysis duration, hemodialysis, lactic acidosis, metformin-associated lactic acidosis, metformin-induced lactic acidosis, suicidal attempt, timing of dialysis initiation

## Abstract

Metformin-induced lactic acidosis (MILA) is a rare but potentially life-threatening condition. It is mainly related to metformin accumulation secondary to overdose or severe kidney failure. We report a case of MILA in a 17-year-old non-diabetic female, who attempted suicide with a high dose of metformin (30 tablets of metformin 500 mg). On arrival, she was fully conscious with normal vital signs. She was admitted to the intensive care unit for close observation. Her laboratory investigations showed development of high anion gap metabolic acidosis, worsening lactatemia, and a drop in blood pressure despite supportive measures. She also required sedation for severe agitation. Intermittent hemodialysis was initiated when the lactate blood level reached 18 mmol/L, accompanied by a pH of 7.1, and was maintained until a target lactate level of less than 4 mmol/L was achieved, which occurred after eight hours. Her clinical condition showed improved mental status by the end of the dialysis session. She left the hospital after a few days in excellent clinical condition. This case highlights a rare situation of suicidal attempts using a high dose of metformin by a non-diabetic minor patient. It also highlights the importance of urgent dialysis if supportive measures fail to mitigate the potential life-threatening MILA condition.

## Introduction

Metformin is primarily known as a widely used antidiabetic medication that decreases hepatic glucose production through reduction of gluconeogenesis and enhances insulin sensitivity in peripheral tissues [1]. Metformin also has other off-label indications, among which we can cite polycystic ovary syndrome (PCOS), prediabetes/insulin resistance, and gestational diabetes mellitus [2]. While it is a safe drug with proven efficacy and cardiovascular benefits, it can also increase plasma lactate levels due to its effects on mitochondrial function. Therefore, one of its rare but potentially life-threatening adverse effects is metformin-associated lactic acidosis (MALA). Although the incidence is low, estimated at fewer than 10 cases per 100,000 patient-years, MALA carries a high mortality rate [3].

Lactic acidosis in the context of metformin use is most often precipitated by conditions that impair lactate clearance or promote lactate production, such as renal dysfunction, sepsis, hypoperfusion, or hepatic impairment [4]. Metformin accumulation in these settings can inhibit mitochondrial respiration, leading to elevated lactate levels and a severe anion gap metabolic acidosis [5].

In diabetic patients on metformin, risk factors for MALA include impaired kidney and liver functions, sepsis, or hypoxia [4]. Metformin-induced lactic acidosis (MILA) is a subset of MALA in which metformin alone is the primary cause of lactic acidosis. In this situation, metformin accumulates secondary to overdose or severe renal failure.

While the terms MALA and MILA are sometimes used interchangeably, MILA is typically reserved for cases where metformin is the primary driver of lactic acidosis, such as in intentional overdose. It is therefore a type B lactic acidosis, not related to hypoxia.

In this report, we present a case of MILA in a 17-year-old non-diabetic female, following a suicidal attempt with metformin. The patient was on metformin for PCOS, with no other significant comorbid conditions contributing to lactic acidosis. This case also highlights a rare event of suicide in a minor patient with a medicine that may seem harmless. We also reviewed some aspects of MILA related to its incidence and management.

## Case presentation

**Figure 1:**
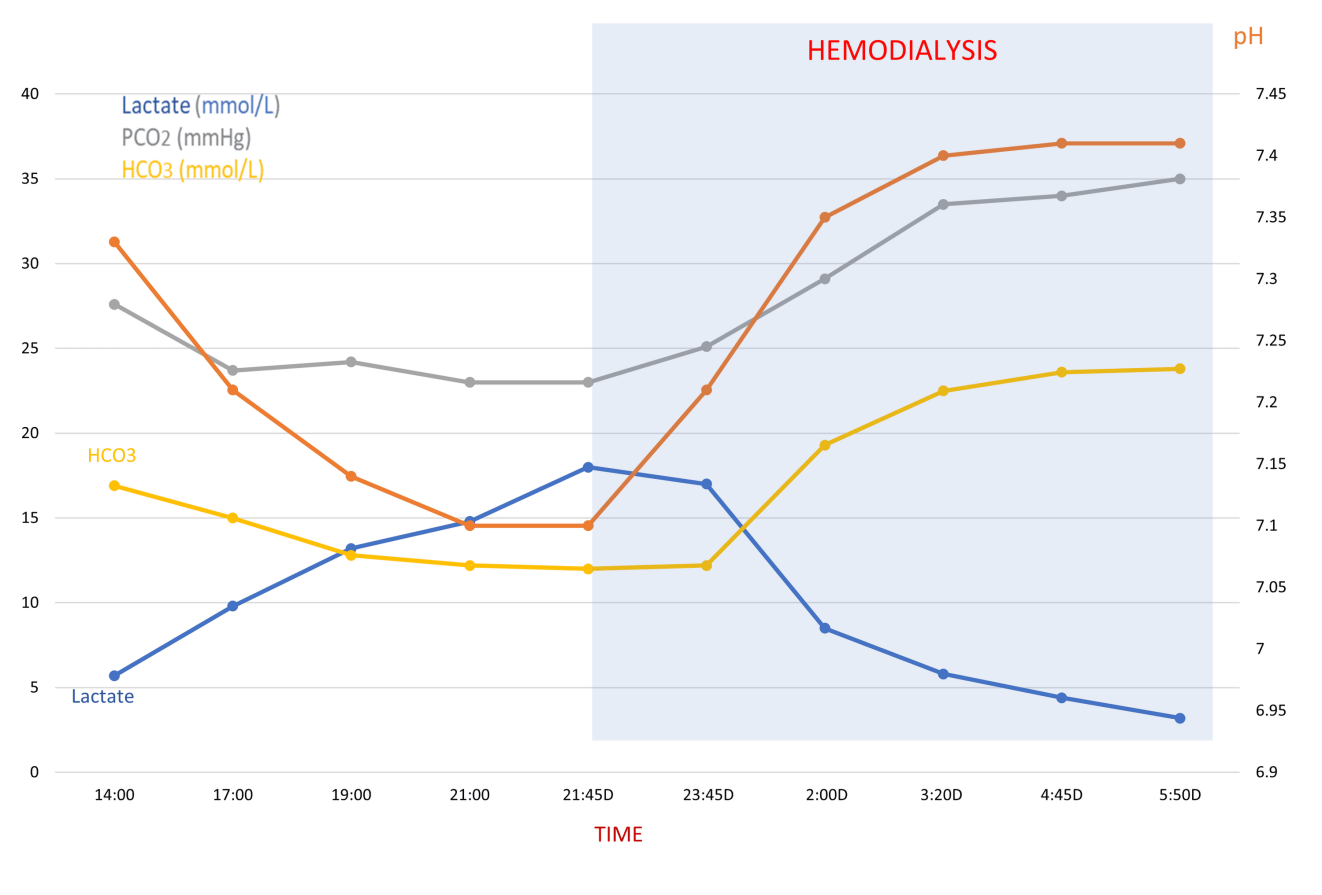
Progress of biochemical parameters. Blue area represents the dialysis period. PCO2: partial pressure of carbon dioxide; HCO3: bicarbonate.

A 17-year-old non-diabetic female was brought to the emergency department by relatives who reported ingestion by the patient of approximately 30 tablets of metformin 500 mg in a suicidal attempt. The patient was regularly using metformin prescribed for PCOS. Apart from that, the patient had no chronic illness and was not on any other medications. On arrival, she was fully conscious, oriented, and hemodynamically stable. She reported nausea and abdominal pain and had vomited twice. Her initial vital signs included a normal blood pressure (115/70 mmHg), heart rate of 96 beats per minute, temperature of 36.8°C, and oxygen saturation of 99% on room air. Her Glasgow Coma Scale (GCS) score was 15/15. Initial laboratory investigations done at 14:00 hours shortly after presentation to the emergency department showed normal serum electrolytes, normal kidney and liver function tests, as well as the complete blood count. Plasma glucose was normal (5.2 mmol/L). In view of the potential metformin toxicity, an arterial blood gas (ABG) was performed and revealed high anion gap (AG) metabolic acidosis (AG = 14.1) with pH at 7.3 and high lactate level (5.7 mmol/L). The patient was admitted to the intensive care unit (ICU) for close monitoring. During the first few hours of admission, a progressive drop in blood pressure was noticed, reaching 85/50 mmHg, which dictated the start of noradrenaline infusion and intravenous fluid resuscitation. She was also sedated as she became increasingly agitated and uncooperative after a few hours of ICU admission. The patient repeatedly attempted to climb out of bed and remove her monitoring devices, posing a risk of self-harm. Her agitation was attributed to a combination of metabolic derangements and the evolving clinical condition. Other supportive measures included the intravenous infusion of sodium bicarbonate. Despite these measures, the anion gap metabolic acidosis did not show improvement. Serial ABG analyses clearly showed worsening metabolic acidosis with a pH down to 7.1 and a persistent increase in lactate level, which peaked at 18 mmol/L. Therefore, an urgent intermittent dialysis was initiated. It was continued with the target of achieving a serum lactate level below 4 mmol/L. This latter target was obtained after eight continuous hours of dialysis (lactate = 3.2 mmol/L). Her clinical condition and mental status improved even before the end of the dialysis session. Noradrenaline was tapered and discontinued following completion of hemodialysis. She left the hospital after a few days in excellent clinical condition. Table 1 and Figure 1 show the progress of biochemical parameters from admission till the end of dialysis, demonstrating a clear correlation between dialysis initiation and metabolic recovery.

**Table 1:** Value of biochemical parameters from admission till the end of dialysis. The progress of the parameters during intermittent hemodialysis is shown below the empty row. In brackets are the normal values for lactate, pH, partial pressure of carbon dioxide (PCO2), and bicarbonate (HCO3).

Time	Lactate (0.3-0.8mmol/l)	pH (7.35-7.48)	PCO2 (32-48 mmHg)	HCO3 (22-28.3 mmol/l)
14:00	5.7	7.33	27.6	16.9
17:00	9.8	7.21	23.7	15
19:00	13.2	7.14	24.2	12.8
21:00	14.8	7.1	23	12.2
21:45	18	7.1	23	12
23:45	17	7.21	25.1	12.2
2:00	8.5	7.35	29.1	19.3
3:20	5.8	7.4	33.5	22.5
4:45	4.4	7.41	34	23.6
5:50	3.2	7.41	35	23.8

## Discussion

While MALA typically involves comorbid conditions, MILA refers to lactic acidosis caused primarily by metformin overdose, like in our patient with suicidal attempt, or severe accumulation in advanced kidney failure patients while still consuming metformin [4]. In MILA, no other major contributing factors like sepsis or hypoxia are present. Lactic acidosis is directly attributable to metformin toxicity occurring in otherwise healthy individuals [6].

The high metformin plasma concentration that follows causes dose-dependent inhibition of complex I of the mitochondrial respiratory chain, which impairs oxidative phosphorylation, entire shift to anaerobic metabolism, and therefore excess lactate generation from pyruvate [7]. It also suppresses hepatic gluconeogenesis by inhibiting the mitochondrial glycerol-3-phosphate dehydrogenase, leading to the inability of the liver to recycle lactate to glucose [8]. The clinical result is a systemic metabolic disruption, profound lactic acidosis (pH < 7.0 is common), and multi-organ failure, if untreated.

Metformin is a small water-soluble molecule, minimally protein-bound and therefore easily dialyzable [9,10]. However, metformin clearance by intermittent hemodialysis has been shown to be far better than with continuous renal replacement therapy (200 mL/minute and 50 mL/minute, respectively) [11,12]. Therefore, it must be highlighted that intermittent hemodialysis is the preferred modality as it is highly efficient in correcting acidemia and removing metformin; unless the patient is hemodynamically unstable, then continuous renal replacement therapy is the best alternative [9].

The Extracorporeal Treatments in Poisoning (EXTRIP) Workgroup has recommended the institution of extracorporeal removal when any one of the following conditions of severity is met: lactate > 20 mmol/L, pH ≤ 7.0, or failure of standard supportive measures (e.g., use of bicarbonate infusion) [10]. It has also been suggested that dialysis may be indicated if lactate concentration > 15-20 mmol/L, pH < 7.0-7.1, and particularly if one of the following comorbid conditions is present: shock, impaired kidney function, liver failure, and decreased level of consciousness [10]. In our patient, the indication of starting intermittent hemodialysis was based on the second aspect of EXTRIP criteria, with a lactate level of 18, a pH of 7.1, and the presence of decreased level of consciousness. Moreover, in a 10-year retrospective analysis (2010-2019), the National Poisons Information Service in the United Kingdom found that serum lactate concentration did not correlate with arterial pH and that it represents an independent marker of poisoning severity [13].

On the other hand, measuring metformin levels before prescribing extracorporeal treatment does not appear clinically important. Indeed, metformin toxicity cannot be excluded even if levels are not high [10].

The recommendations from EXTRIP stipulate that extracorporeal treatment can be stopped when the lactate concentration falls below 3 mmol/L, and the pH corrects to at least 7.35, although repeated monitoring is required to detect rebound of MALA due to redistribution of metformin from tissues to the intravascular space [10]. Our patient needed only one prolonged session of hemodialysis (eight hours) to normalize the pH and decrease the lactate level from 18 to 3.3. Repeated biochemistry parameters the day after were within normal range. In their 10-year retrospective analysis of MALA, Seidowsky et al. reported a total of 42 cases, among which 13 were cases of voluntary intoxication with metformin [14]. No death was observed in intentional overdose patients, but in incidental overdose patients, the mortality was high (48.3%). The factors significantly associated with mortality were multiorgan dysfunction, pH, plasma lactate, and prothrombin activity. They reported normalization of metformin levels after a cumulative hemodialysis duration of 15 hours [14].

Two meta summaries of MALA case reports and case series analyzed 242 and 253 patients, respectively [15,16]. In both studies, the most frequent symptom was conscious level deterioration. The mortality rate was similar, i.e., 19.8 % vs. 17.2%, respectively. Regarding metformin overdose and MILA, there is currently no epidemiologic data, but only case reports and a few case series are available in the literature. Among these, a few are of interest in relation to suicidal attempt with metformin. Looking at their data from 1995 until 2003, a poison center in Germany recorded six cases of MILA, with mortality in four of them [17]. In a data analysis by the Western France Poison Control Centre, out of 382 cases of metformin poisoning identified from September 1999 to September 2016, 63 patients developed MALA, including 19 cases of MILA related to self-poisoning [18]. Avcı et al. reported a series of five cases of suicidal attempt with metformin observed in a large tertiary center in Türkiye from January 2011 to March 2012 [19]. However, the distinction between MALA and MILA is not always noted in the published literature [10].

Looking at the outcome in relation to blood pH, lactate, and metformin levels, the available data in the literature tend to consider these biochemical parameters rather poor predictors of mortality in patients with MALA [14,20]. We therefore believe that the indication for starting dialysis should also take into consideration the clinical presentation and the speed of clinical deterioration, particularly of the neurological status.

## Conclusions

In conclusion, this case highlights a rare condition of MILA following a suicidal attempt with a high dose of metformin in a minor patient. Our case strengthens the point that early ICU admission of apparently stable patients suspected of metformin overdose (as in our case) is very important and should be part of the management of MILA. Close follow-up of pH, lactate level, and the patient's condition will dictate the start of intermittent hemodialysis and ease the prognosis of this serious condition. As MILA carries a high mortality risk and considering the widespread and increasing use of metformin globally, timely recognition and management of MILA/MALA are critical.
